# The influence of different fever definitions on diagnostics and treatment after diagnosis of fever in chemotherapy-induced neutropenia in children with cancer

**DOI:** 10.1371/journal.pone.0193227

**Published:** 2018-02-20

**Authors:** Stéphanie Wagner, Eva K. Brack, Eveline Stutz-Grunder, Philipp Agyeman, Kurt Leibundgut, Oliver Teuffel, Roland A. Ammann

**Affiliations:** 1 Department of Pediatrics, Inselspital, Bern University Hospital, University of Bern, Bern, Switzerland; 2 Department of Infectious Disease and Cancer Research, Children´s Hospital Zurich, University of Zurich, Zurich, Switzerland; 3 Department of Pediatric Oncology, Children´s Hospital Zurich, University of Zurich, Zurich, Switzerland; 4 Division of Oncology, Medical Services of the Statutory Health Insurance Baden-Württemberg, Tübingen, Germany; 2nd medical school of Charles University, CZECH REPUBLIC

## Abstract

**Background:**

There is no evidence-based definition of the temperature limit defining fever (TLDF) in children with neutropenia. Lowering the TLDF is known to increase the number of episodes of fever in neutropenia (FN). This study aimed to investigate the influence of a lower versus standard TLDF on diagnostics and therapy.

**Methods:**

In a single pediatric cancer center using a high standard TLDF (39°C tympanic-temperature) patients were observed prospectively (NCT01683370). The effect of applying lower TLDFs (range 37.5°C to 38.9°C) versus 39.0°C on these measures was simulated in silicon.

**Results:**

In reality, 45 FN episodes were diagnosed. Of 3391 temperatures measured, 193 were ≥39.0°C, and 937 ≥38.0°C. For persisting fever ≥24 hours, additional blood cultures were taken in 31 (69%) episodes in reality. This number decreased to 22 (49%) when applying 39.0°C, and increased to 33 for 38.0°C (73%; plus 11 episodes; plus 24%). For persisting fever ≥48 hours, i.v.-antibiotics were escalated in 25 (56%) episodes. This number decreased to 15 (33%) when applying 39.0°C, and increased to 26 for 38.0°C (58%; plus 11 episodes; plus 24%). For persisting fever ≥120 hours, i.v.-antifungals were added in 4 (9%) episodes. This number increased to 6 (13%) by virtually applying 39.0°C, and to 11 for 38.0°C (24%; plus 5 episodes; plus 11%). The median length of stay was 5.7 days (range, 0.8 to 43.4). In 43 episodes with hospital discharge beyond 24 hours, applying 38.0°C led to discharge delay by ≥12 hours in 24 episodes (56%; 95% CI, 40 to 71), with a median delay of 13 hours, and a cumulative delay of 68 days.

**Conclusion:**

Applying a low versus standard TLDF led to relevant increases of diagnostics, antimicrobial therapy, and length of stay. The differences between management in reality versus simply applying 39.0° as TLDF reflect the important impact of clinical assessment.

## Introduction

In children with cancer, fever in chemotherapy-induced severe neutropenia (FN) is the most frequent potentially lethal complication and the leading cause of emergency hospitalization [1]. Empirical therapy with broad-spectrum antibiotics, usually given intravenously in an in-house setting, are routine measures for pediatric FN. They have led to a reduction of the FN associated mortality below 1% in developed countries [2]. However, there is no international consensus on temperature-limits defining fever (TLDF) for children with chemotherapy-induced severe neutropenia, despite its relevance for the clinical diagnosis and management of FN [1,3,4]. A review of the current literature shows a wide range of TLDF used clinically from 37.5°C to 39.0°C [5–7]. Through this inconsistency between different centers, the diagnosis of FN is made at varying temperatures. At our center in Bern, with a historically established high TLDF of 39°C ear-temperature, it has recently been shown that this high TLDF is efficacious in reducing the number of FN diagnosis and thus overtreatment. Lowering this limit would have led to additional FN diagnosis [7].

Not only the diagnosis of FN and the initial therapy are influenced by the TLDF applied, but also the clinical measures of diagnostics and treatment during the whole hospitalization for FN are determined by the TLDF applied. Those measures include for example the number of blood cultures taken, the time-point of escalating empirical antibiotic therapy to a broader spectrum, or the time point of adding on empirical antifungal treatment in case of persisting fever [1]. Moreover, it influences the time point of hospital discharge and thus the length of hospitalization. These measures not only have a direct impact on the individual patient and its quality of life but also on resource utilization and treatment costs [8].

Based on the historically established clinical use of a high TLDF of 39°C in Bern [7,9] this prospective single-center study aimed to assess the influence of virtually lowering the TLDF of 39°C ear-temperature on diagnostic measures, time-points of treatment escalation and hospital discharge after FN diagnosis.

## Patients and methods

### Study design

This prospective observational study was conducted in a single pediatric cancer center that routinely uses a high TLDF (39.0°C tympanic temperature, Limit_Standard_). The intervention of virtually lowering the TLDF and its effects on FN related measures were simulated *in silicon* using commercially available software. Therefore, diagnosis of FN, initial treatment of the study patients and their clinical management after FN diagnosis were not influenced by study participation.

The study was conducted in accordance with the Declaration of Helsinki. The Institutional Review Board (Ethikkommission der Universitätskinderkliniken Bern, “Gesuch 1205”) had approved the study protocol and the trial had been registered at www.clinicaltrials.com (NCT01683370) before starting patient accrual. Written informed consent was obtained from the patients, if able to judge, and from their legal guardians prior to study entry.

The effects of virtually lowering the TLDF on the rate of FN diagnosis itself have been published elsewhere [7].

### Patients

Patients aged 1 to 17 years with cancer who were treated at the Department of Pediatrics, University of Bern, Inselspital, Bern, Switzerland, and who required chemotherapy for ≥2 months at time of recruitment were eligible.

Patients were off study when informed consent was withdrawn and when chemotherapy was completed (≥2 weeks after last dose and absolute neutrophil count (ANC) >0.5 G/L). Patients were recruited from August 11, 2012 to May 31, 2013, while follow-up was closed on August 07, 2013.

### Routine clinical management, including FN

Patients were treated with chemotherapy, including myeloablative therapy followed by autologous peripheral stem cell transplantations, or multimodal therapy, according to internationally established protocols. Except for standard *Pneumocystis jirovecii* prophylaxis, no primary antimicrobial prophylaxis was used [10,11]. Secondary antifungal or antiviral prophylaxis was introduced after repeated infections.

FN was diagnosed when a patient with severe chemotherapy-induced neutropenia developed fever. Fever was defined as a single temperature of ≥39.0°C (Limit_Standard_), measured in the ear by infrared tympanic thermometry using the commercially available Braun Thermoscan 5 (IRT 4520; Braun GmbH, Kronberg, Germany) [12]. This TLDF corresponds to 39.1°C core temperature, and to 38.4°C axillary temperature (Nimah, PCCM, 2006). Severe neutropenia was defined as an ANC ≤0.5 G/L or ≤1.0 G/L and expected to decline. If clinically indicated, the treating physician was free to diagnose FN at lower temperatures [2,9,13].

Routine clinical measures after FN diagnosis included emergency hospitalization, taking blood cultures (BC) from the central venous access device before starting empirical intravenous broad-spectrum antibiotics, usually ceftriaxone plus amikacin and antipyretics [14]. Further details on clinical management have been published [2,9].

For persisting fever despite treatment, the following clinical rules were usually applied: every 24 hours additional BC were taken; after 48 hours the empirical intravenous antibiotics were escalated, usually to meropenem and vancomycin, for a broader antimicrobial coverage; and after 120 hours, empirical intravenous antifungals were added [15]. Patients were discharged when they had been afebrile for ≥48 hours, were clinically well, had a rising leukocyte count and/or ANC irrespective of absolute values, and had negative blood cultures.

### Data management, outcome measures and analysis

Results of all temperature measurements and information on the diagnostic and therapeutic measures after FN diagnosis studied here were extracted from patients’ charts and directly entered into Excel-spreadsheets by an experienced pediatric oncology nurse. This information was checked for plausibility and, in case of doubt, conformity with patient charts by two senior pediatric oncologists (E.S.-G., R.A.A.).

The analysis was performed by a pediatric trainee (S.W.) under supervision of the senior author (R.A.A). The following outcome measures, all related to persistent fever as described above, were analyzed: episodes with at least one additional BC taken after start of antibiotics; with escalation of antibiotic coverage, with start of empirical antifungals, and with hospital discharge delayed ≥12 hours because of fever. The measures determined in reality were compared with measures virtually determined by strictly applying the rules for persisting fever described above, using Limit_Standard_ (TLDF 39.0°C), and different settings for Limit_Low_, (TLDF ranging from 37.5°C to 38.9°C) [7,16]. Four FN episodes that had been diagnosed below 39.0°C for clinical reasons and in which 39.0°C was never reached were excluded from the analysis of delayed hospital discharge, as the in silico simulation relied on FN diagnosis made at ≥39.0°C [7]. One further FN episode was excluded from this specific analysis due to early discharge before 24 hours because of a low risk of complications [17–19].

Compared to the previously published results on the primary study aims [7], two additional FN episodes are reported here. This results from splitting up one FN episode with continued oral antimicrobial therapy for osteomyelitis over six months into three separate FN episodes. Formally, this episode fulfilled the study criteria for a single episode because of continued antimicrobial therapy, and was treated as such for the analysis focused on FN diagnosis [7]. In reality, however, the patient was hospitalized three times for FN, with outpatient periods exceeding one month between hospitalizations. Analyzing these hospitalizations as separate FN episodes seemed to be more adequate for the outcomes reported here.

### Statistics

Due to non-normally distributed data, median, interquartile range (IQR), and range were calculated. Proportions with exact 95% confidence intervals (CI) were calculated [20]. The sample size had been determined to result in an adequate power for analysis of the primary efficacy aim of the study, the number of FN episodes diagnosed [7], and thus was not adapted to fulfill specific power requirements for the secondary aims described here.

The *in silicon* simulation by virtually applying different TLDFs was performed in *Excel 2010* spreadsheets (Microsoft, Redmond, Washington, USA), and statistical analyses in *R 3*.*2*.*3* (R Foundation for Statistical Computing, Vienna, Austria). *P*-values <0.05 were considered significant.

## Results

### Patients, FN episodes, temperature measurements and complete blood counts (CBC)

Patients were recruited from August 2012 to May 2013. Of 40 patients potentially eligible, 39 (98%) gave informed consent and thus participated in the study. Median age at recruitment was 7.4 years (range, 1.2 to 16.7), and 16 (41%) were girls. The distribution of cancer diagnosis and further details about the therapy and interventions have been published elsewhere [7].

During a cumulative duration of 289 months (24 years) of chemotherapy, 45 FN episodes were diagnosed in 20 patients (maximum, 6 episodes per patient). Of these, 12 (27%) episodes had been diagnosed by the treating physician at temperatures below 39.0°C for clinical reasons [7].

**Fig 1:**
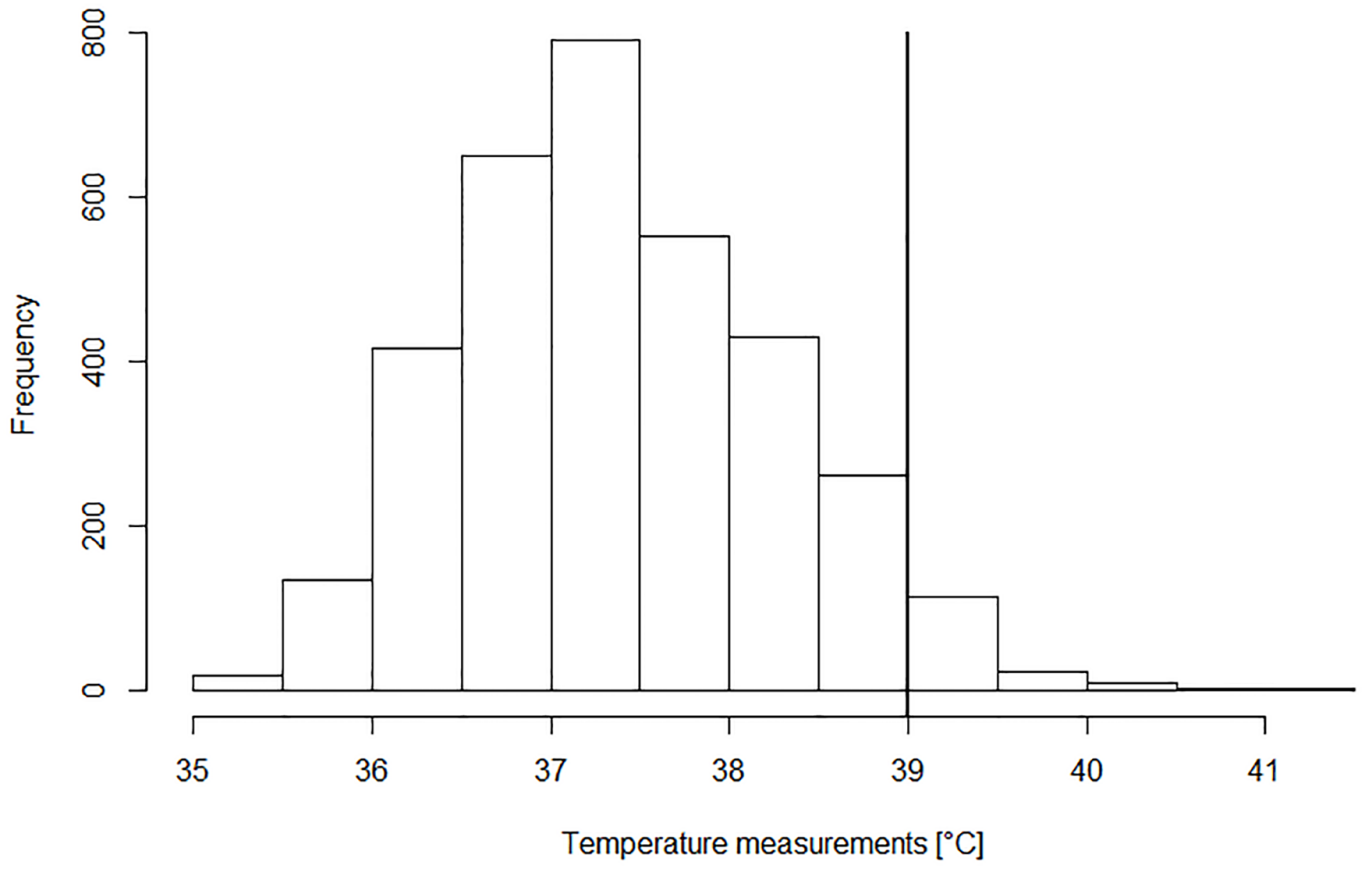
Temperature measurements during FN (n = 3391).

**Fig 2:**
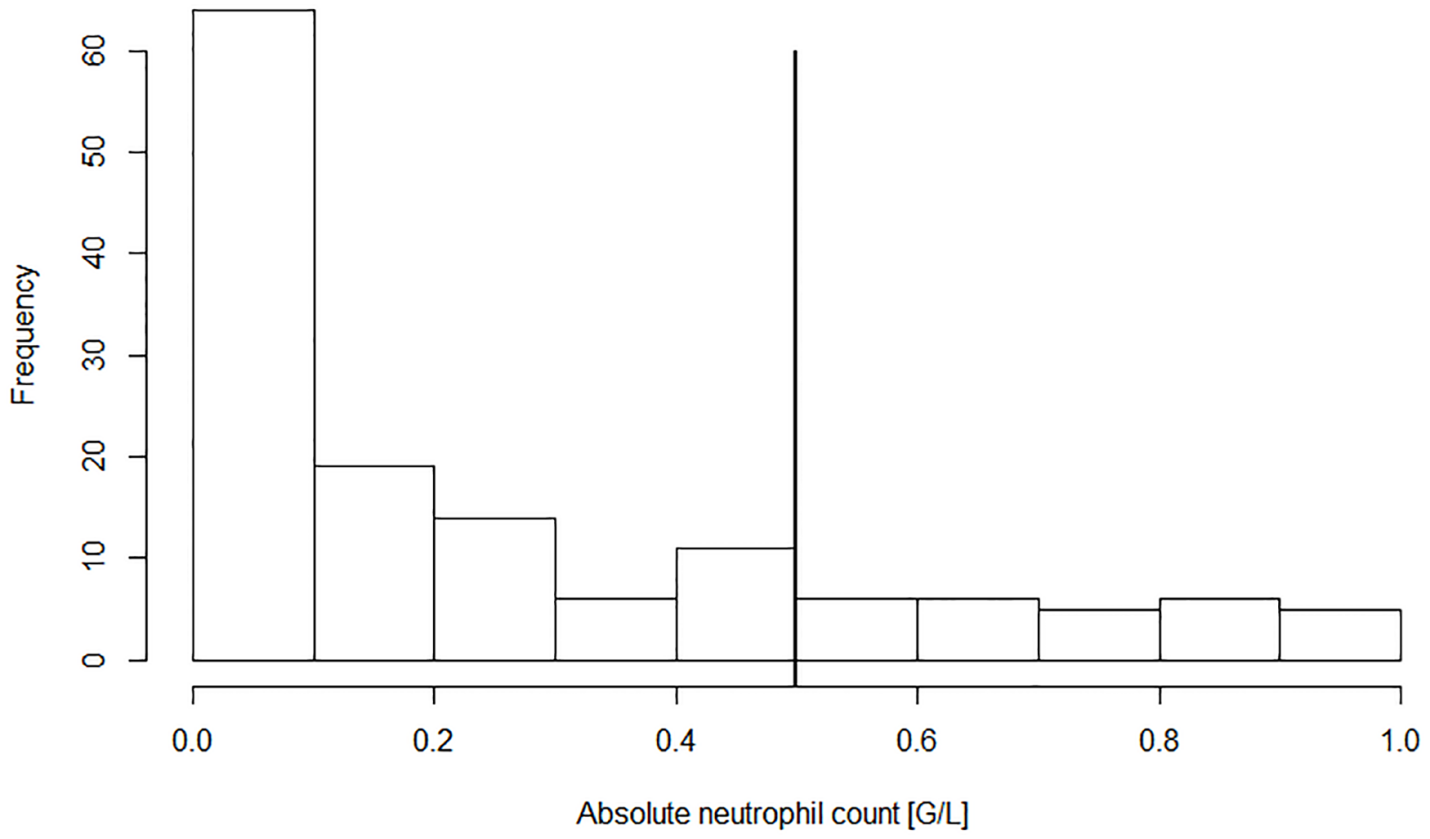
Absolute neutrophil counts in blood counts during FN (n = 295). Displayed are the absolute neutrophil counts up to 1 G/L.

During the FN episodes (cumulative duration, 329 days) 3391 temperature measurements were recorded (median, 56 per episode; IQR, 40 to 83; range, 7 to 401). The median temperature was 37.3°C (IQR, 36.8°C to 38.0°C; range, 35.0°C to 41.2°C) and 193 measurements (5.7%) were ≥39.0°C (Limit_Standard_, Fig 1). Furthermore, 318 CBCs were performed (median, 6 per episode; IQR, 5 to 7; range, 2 to 32). The ANC was ≤0.5 G/L in 208 CBCs (65%), 0.5 to 1.0 G/L in 29 (9%), >1.0 G/L in 58 (18%), and unknown in 23 (7%, Fig 2).

### Additional blood cultures after start of antibiotics

**Fig 3:**
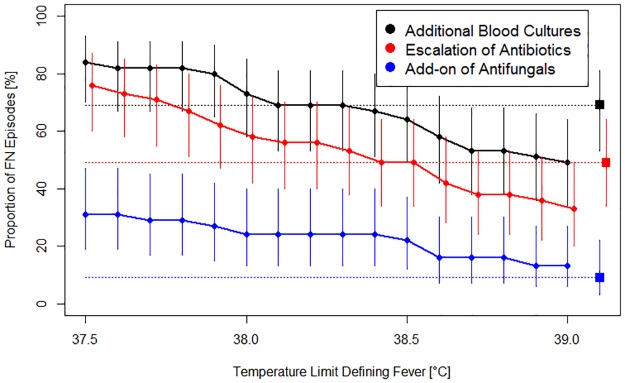
Proportion of FN episodes with modifications of management for persisting fever, in reality and virtually according to different TLDF. Displayed are the proportions, plus their 95% CI as vertical lines, of FN episodes with additional blood cultures taken, with an escalation of antibiotics and with adding on antifungals, respectively, for persisting fever. Squares indicate management in reality, and circles indicate management simulated, strictly applying Limit_Standard_ and different Limit_Low_.

In reality, 122 BC were taken in total, with a median of 2 BC per FN episode (range, 1 to 11). Of these, 45 were BC taken at the initial presentation for FN, and 77 were additional BC taken after start of antibiotics in 31 of 45 episodes (69%; 95% CI, 53% to 82%), usually for persisting fever. Virtually applying Limit_Standard_ (39.0°C) as TLDF for persisting fever, 43 additional BC in 22 episodes (49%; 95% CI, 34% to 64%; minus 9 episodes compared to reality) remained. Virtually applying a range of Limit_Low_, this number increased to 68 additional BC in 29 episodes (64%; minus 2) for 38.5°C, to 103 additional BC in 33 episodes (73%; plus 2) for 38.0°C, and to 148 additional BC in 38 episodes (84%; plus 7) for 37.5°C (Fig 3, Table 1).

**Table 1:** Management of FN episodes diagnosed in reality, and virtually at different temperature limits defining fever.

Type of analysis, item	FN diagnosed in reality	FN virtually diagnosed using limit_Standard_ or limit_Low_			
		39.0°C	38.5°C	38.0°C	37.5°C
FN episodes with additional BC taken^1^	31	22	29	33	38
Number of additional BC taken^1^	77	43	68	103	148
FN episodes with escalation of empirical antibiotics^2^	22	15	22	26	34
Median time-point of escalation of empirical antibiotic’s (hours)	49	74	69	56	53
FN episodes with addition of antifungal therapy^3^	4	6	10	11	14
Number of antifungal therapies added^3^	5	10	15	19	23
Median time of addition of antifungal therapy (hours)	177	161	138	129	127
FN episodes with delayed hospital discharge ≥12h	-	0	12	21	32
Median delay of hospital discharge (hours)	-	0	1	13	37
Cumulative delay of hospital discharge (hours)	-	0	832	1295	2312

FN, fever in neutropenia; BC, blood cultures.

^1^for persisting fever ≥ 24h after start of antibiotics;

^2^for persisting fever ≥ 48h after start of antibiotics;

^3^for persisting fever ≥ 120h after start of antibiotics

Of the 122 BC, 10 were reported positive, but 1 was considered false positive (contamination with *Micrococcus* species). Thus, in 9 of the 45 FN episodes (20%; 95% CI, 10% to 35%), bacteremia was detected.

The species detected were: *Klebsiella pneumonia* in 2 episodes, *Actinomyces odontolyticus* plus *S*. *viridans* in one episode and *Enterococcus faecium*, *Staphylococcus aureus*, coagulase-negative *Staphylococcus*, *Streptococcus viridans*, *Fusobacterium* species and *Moraxella catarrhalis* in one episode each.

In 6 FN episodes the initial BC was reported positive. In 3 episodes the initial BC showed no bacterial or fungal growth but a BC taken after start of antibiotic therapy revealed bacteremia (one each with coagulase-negative *Staphylococcus*, E. *faecium* and S. *aureus*). The temperature at taking the respective BC was below Limit_Standard_ in two episodes (38.6°C and 37.9°C, respectively), and ≥Limit_Standard_ in one.

### Escalation of empirical antibiotics

In reality, intravenous antibiotics were given for a median of 5.7 days (range, 1 to 43). They were escalated in order to achieve broader coverage after a median of 49 hours (range, 7 to 112) in 22 (49%; 95% CI, 34% to 64%) FN episodes, usually for persisting fever ≥48 hours. Virtually applying Limit_Standard_ (39.0°C) as TLDF for persisting fever resulted in escalation after a median of 74hours in 15 episodes (33%; 95% CI, 20% to 49%; minus 7 episodes compared to reality). Virtually applying a range of Limit_Low_ increased the number of such episodes to 22 (49%; 95% CI, 34% to 64%, as in reality) for 38.5°C, to 26 (58%; 95% CI, 42% to 72%, plus 4) for 38.0°C, and to 34 (76%; 95% CI, 60% to 87%, plus 12) for 37.5°C (Fig 3, Table 1).

### Addition of empirical antifungal therapy

In reality, an intravenous empirical antifungal therapy was added after a median of 177 hours (7.4 days; range, 2.6 to 18.5 days) in 4 (9%; 95% CI, 2% to 21%) FN episodes, usually for persisting fever ≥5 days. Virtually applying Limit_Standard_ (39.0°C) as TLDF for persisting fever resulted in the addition of an intravenous empirical antifungal therapy in 6 episodes (13%; 5% to 27%; plus 2 episodes compared to reality) after a median of 161 hours (6.7 days). Virtually applying a range of Limit_Low_, increased the number of such episodes to 10 (22%; plus 6) for 38.5°C, to 11 (24%; plus 7) for 38.0°C, and to 14 (31%; plus 10) for 37.5°C. (Fig 3, Table 1). One further patient with AML had received an intravenous antifungal therapy with caspofungin intravenously (iv) at presentation with FN in addition to the empiric antibiotics as *Candida albicans* had been cultured 6 days before FN diagnosis from both skin and oral swabs.

### Delayed discharge

**Fig 4:**
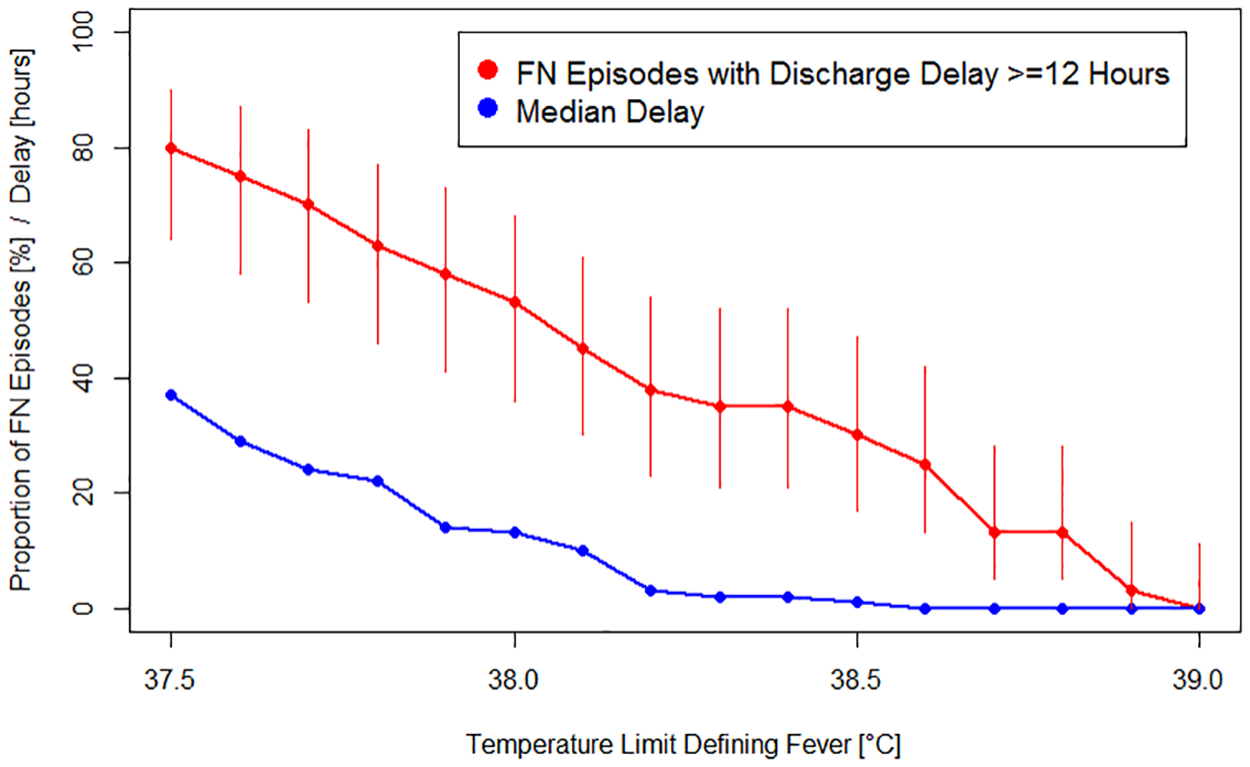
Discharge delay according to TLDF. Displayed are proportions of FN episodes (filled red circles) with their 95% CI (vertical lines) and the median delay of all episodes in hours (filled blue circle).

In the entire set of 45 FN episodes, the median duration of hospitalization was 5.7 days (IQR, 3.1 to 8.8; range, 0.8 to 43). In the 40 episodes analyzed for delayed discharge (at least one temperature measurement ≥ 39.0°C, no early discharge < 24 hours), the median duration was 0.8 hours (IQR, 0 to 22.6, range, 0 to 172.4) when applying a TLDF of 38.5°C instead of 39.0°C. In these 40 episodes, virtually applying a range of different Limit_Low_, resulted in 12 FN episodes with discharge delay ≥ 12 hours for 38.5°C (30%; median delay 1 hour; IQR, 0 to 22; range, 0 to 172); in 21 episodes for 38.0°C (53%; median delay 13 hours; IQR, 1 to 35; range, 0 to 172), and in 32 episodes for 37.5°C (80%; median delay 37 hours; IQR, 13 to 75; range, 0 to 286) (Fig 4, Table 1).

## Discussion

The efficacy of a high versus low TLDF in patients with chemotherapy induced neutropenia has been shown to significantly reduce the number of emergency calls for fever, of performed CBCs and of overall FN diagnosis [7]. The results presented here further demonstrate its efficacy by significant and clinically relevant reductions of both diagnostic (additional BCs) and therapeutic measures (escalation of empirical antibiotics, addition of empirical antifungal therapy, length of hospital stay) after the diagnosis of FN has been made. These reductions, in return, stand for less side effects, less treatment costs and a better quality of life for the patient and his family [8,21].

Strictly applying a high TLDF of 39.0°C, irrespective of the clinical condition, would have halved the number of additional BC taken after start of antimicrobial therapy. As one third of the bacteremia was detected in such additional BC, both a strict application of this Limit_Standard_ or a further increase of this TLDF would lead to delayed or missed diagnoses of bacteremia in a relevant proportion of FN, with potentially detrimental clinical consequences. In reality, the treating physician was free to ask for additional BC below this Limit_standard_ when clinically indicated, which reduced this risk and maintained clinical safety. As previously shown, half of the reported adverse events (AEs) were detected in episodes diagnosed below 39.0°C; so within an oncology center, it has to be assured, that every treating physician is aware about this fact and that the Limit_standard_ should be considered as a guidance and not as a rule carved in stone. This once more, underlines the importance of clinical judgement overrating a predefined TLDF when needed.

Taken together, in reality a relevant number of FN episodes was diagnosed below Limit_Standard_ [7] and diagnostics were frequently escalated by additional BC below Limit_Standard_ as well. In line with this, empirical antibiotics were as well frequently escalated below Limit_Standard_ for clinical reasons. In sharp contrast to these three consistent observations, escalation of therapy by adding empirical antifungals was less frequently observed in reality than defined by strict application of Limit_Standard_. Interestingly, this inversely oriented discrepancy reflects the importance of clinical judgment as well. Here the treating physician refrained from adding antifungals in patients without high risk for invasive fungal infection and in good general condition despite prolonged fever ≥Limit_Standard_, consistent with past and current guidelines [1,15].

In one third of FN episodes, lowering the TLDF from 39.0° C to 38.5° C would have resulted in a delay in hospital discharge exceeding 12 hours.

The calculated discharge delays are additionally underestimated due to incomplete recordings beyond 48 hours from the last temperatures ≥39.0°C because, in reality, patients were discharged. Correspondingly, lowering the TLDF would have resulted in relevant cost increase.

To our knowledge, this is the first prospective, though purely observational, study of the influence of different fever definitions on diagnostics and treatment after the diagnosis of fever in chemotherapy-induced neutropenia in children with cancer.

The findings of this study are based on large numbers of prospectively recorded temperature measurements. The in silicon simulation allowed for a non-interventional study. This study critically relied on the high standard TLDF of 39.0°C used in Bern. A reverse design, i.e., virtually assessing the impact of higher TLDFs in centers using low or medium TLDFs, is made impossible by the routine application of antipyretics after FN diagnosis. Potential limitations are its single center design. Besides, the rather small sample size, powered for efficacy testing of the primary outcome, FN diagnosis [7], precluded meaningful assessment of safety aspects. Currently, the randomized controlled multicenter Swiss Pediatric Oncology Group (SPOG) 2015 FN Definition trial, powered to assess the safety of a high (39.0°C) versus low (38.5°C) TLDF, is recruiting patients (NCT02324231).

In conclusion, this study shows that a high versus low TLDF is efficacious in reducing diagnostic measures, both antibacterial and antifungal antimicrobial therapy and length of hospital stay in children and adolescents with FN during chemotherapy for cancer. Significant and clinically relevant differences in all these measures have been shown for a TLDF of 39.0°C versus 38.5°C. Applying a high TLDF thus may decrease treatment-related side effects and costs and increase the quality of life. The differences between the clinical management found in reality versus the virtual management based on strictly applying Limit_Standard_ underlines the importance of clinical judgement by experienced pediatric oncologists.

## Abbreviations

ANC: Absolute neutrophil count
BC: Blood culture
CBC: Complete blood count
CI: Confidence interval
FN: Fever in (chemotherapy-induced severe) neutropenia
IQR: Interquartile Range
Limit_Low_: Hypothetically applied lower temperature limit defining fever
Limit_Standard_: Standard temperature limit defining fever (≥39.0°C)
TLDF: Temperature limit defining fever
